# Interplay between Skin Sympathetic Nerve Activity and Gut Microbiota, the Neurocardiac Axis in Acute Coronary Syndrome

**DOI:** 10.7150/ijms.126417

**Published:** 2026-08-13

**Authors:** Wei-Chung Tsai, Po Peng, Pei-Syuan Jhou, Yi-Chun Tsai, Wei-Wen Hung, Tien-Chi Huang, Li-Fang Chou, Tsung-Hsien Lin, Chien-Hung Lee, Bin-Nan Wu, Shien-Fong Lin, Wei-Chun Hung

**Affiliations:** 1 Graduate Institute of Clinical Medicine, College of Medicine, Kaohsiung Medical University, Kaohsiung 807378, Taiwan.; 2 Division of Cardiology, Department of Internal Medicine, Kaohsiung Medical University Hospital, Kaohsiung Medical University, Kaohsiung 807377, Taiwan.; 3 Faculty of Medicine, College of Medicine, Kaohsiung Medical University, Kaohsiung 807378, Taiwan.; 4 Precision Sports Medicine and Health Promotion Center, Kaohsiung Medical University, Kaohsiung 807378, Taiwan.; 5 Welgene Biotech. Co., Ltd. Taipei 115603, Taiwan.; 6 Department of Microbiology and Immunology, College of Medicine, Kaohsiung Medical University, Kaohsiung 807378, Taiwan.; 7 Department of Internal Medicine, Kaohsiung Municipal Cijin Hospital, Kaohsiung 805004, Taiwan.; 8 Division of Nephrology, Kaohsiung Medical University Hospital, Kaohsiung Medical University, Kaohsiung 807377, Taiwan.; 9 Division of Endocrinology and Metabolism Department of Internal Medicine, Kaohsiung Medical University Hospital, Kaohsiung Medical University, Kaohsiung 807377, Taiwan.; 10 Department of Public Health College of Health Science, Kaohsiung Medical University, Kaohsiung 807378, Taiwan.; 11 Department of Pharmacology, College of Medicine, Kaohsiung Medical University, Kaohsiung 807378, Taiwan.; 12 Institute of Biomedical Engineering, National Yang Ming Chiao Tung University, Hsinchu 300093, Taiwan.

**Keywords:** skin sympathetic nerve activity, gut microbiota, acute coronary syndrome, neurocardiac axis

## Abstract

Our previous study demonstrated increased skin sympathetic nerve activity (SKNA) in acute coronary syndrome (ACS) patients and its correlation with ventricular arrhythmias. In the current study, we explored specific gut microbial profiles and their relationships with SKNA in ACS patients. Fifteen ACS patients and age- and sex-matched controls were included in this cross-sectional study. Demographic and clinical data were recorded, and SKNA was measured by neuECG. The gut microbial profiles were analyzed by 16S rRNA gene sequencing of the V3-V4 region. Statistically significant differences in alpha diversity (including the Chao 1 index and Faith's phylogenetic diversity) and gut microbial composition were observed between the ACS and control groups. Redundancy analysis and the Mantel test revealed a significant relationship between SKNA and the microbial profiles. The abundance of butyrate-producing bacteria, including *Lachnospira* and *Oscillibacter*, was decreased in the ACS group, whereas the abundance of the genus *Hungatella*, a trimethylamine producer, significantly increased and was positively correlated with the abundance of SKNA. This study is the first to establish an association between SKNA and gut microbial profiles in ACS patients. SKNA may serve as a potential biomarker reflecting the interaction between the nervous system and gut microbiota in ACS.

## 1. Introduction

Acute coronary syndrome (ACS) is among the leading causes of mortality worldwide 1. Hemodynamically significant ventricular arrhythmia (VA) during ACS occurs in approximately 8% of patients and can lead to mortality 2. Sympathetic hyperinnervation caused by ACS can lead to the development of VA. Sympathetic drive leads to increased automaticity, triggered activity, and reentry mechanisms in the heart, all of which can contribute to VA 3,4. Sympathetic overdrive after ACS can provoke further myocardial ischemia, leading to a vicious cycle of VA and sudden cardiac death. Heart rate variability (HRV), heart rate turbulence, and abnormal T wave alternans can be used to assess the risk of death or VA after MI 5,6. neuECG is a well-validated noninvasive method for simultaneously recording electrocardiogram and skin sympathetic nerve activity (SKNA) 7. Increased SKNA is known to precede spontaneous VA in canine models and humans 8-11. We also reported that SKNA increased in ACS patients and correlated with VA in a clinical study 12.

Accumulating evidence has demonstrated the critical roles of gut dysbiosis and the imbalance of microbial metabolites in the development of ACS. A decrease in butyrate-producing bacteria, along with a corresponding reduction in butyrate concentration, is correlated with elevated inflammation, cardiac damage, and increased mortality 13. Trimethylamine N-oxide (TMAO), which is formed from trimethylamine (TMA) generated by the action of gut microbiota, has been reported as a novel prognostic marker for ACS 14. Additionally, the relationships between gut microbiota and circulating metabolites demonstrated by Liu et al. provide a better understanding of the interplay between the host and gut microbiota in atherosclerotic pathogenesis 15. A canine study revealed that TMAO can activate the cardiac sympathetic nervous system (SNS) and worsen ischemic VA via the left stellate ganglion (SG) pathway or central autonomic activation 16. The accumulation of branched-chain amino acids (BCAAs) is an alternative biomarker of cardiometabolic disease 17-19. Pedersen et al. further suggested that elevated serum BCAA levels were attributed to the gut microbiome rather than to dietary intake 20. Recent studies have further highlighted the complex roles of gut microbiota in host metabolic regulation and functional diversity, which may contribute to cardiometabolic diseases through multiple pathways 21, 22.

As mentioned above, gut microbiota have been reported to play a role in ACS and may modulate sympathetic neurons. However, the interplay between the neurocardiac axis and the microbiome in clinical ACS patients is not clearly understood. Considering the above evidence, we conducted this study to test the hypothesis that SKNA is increased in ACS patients compared with controls and that increased SKNA is associated with specific microbes that may play important roles in linking the microbiota, SNS, and ACS.

## 2. Materials and Methods

### 2.1. Patients

This single-center, cross-sectional study was approved by the ethics committee of Kaohsiung Medical University Hospital, and all study subjects provided written informed consent. This study is registered with ClinicalTrials.gov (identifier: NCT03243448). The study participants were enrolled between 2019 and 2021 at Kaohsiung Medical University and Kaohsiung Medical University Hospital. The inclusion criteria were ACS patients and control participants who were 1:1 matched in terms of age and sex. Each group consisted of 15 individuals who were all 20 years of age or older. ACS patients were defined as those with myocardial infarction and unstable angina admitted to the coronary care unit after coronary angiography and/or percutaneous coronary intervention as the standard of care. The enrolled controls were healthy adults who joined the health management program at Kaohsiung Medical University. The exclusion criteria for this study included the use of antibiotics, metformin, or proton pump inhibitors and a diagnosis of diabetes or colon cancer, as all these could have interfered with the results of the microbiota study. Detailed information on medication use is provided in Table S1. These stringent exclusion criteria limited the number of enrolled ACS patients in this study. Demographic and clinical data such as age; sex; body mass index (BMI); clinical factors (hypertension, diabetes, dyslipidemia, and mean arterial pressure); and laboratory data (estimated glomerular filtration rate and serum levels of potassium, triglyceride, low-density lipoprotein (LDL) cholesterol, high-density lipoprotein (HDL) cholesterol, hemoglobin, and hemoglobin A1C) were collected for both the ACS and control groups.

### 2.2. neuECG, SKNA, and HRV Measurements

The detailed methods of neuECG recording have been reported elsewhere 7. In brief, the neuECG used conventional lead I ECG electrodes and equipment with a high sampling rate (10,000 Hz) and wide bandwidth (1-2000 Hz) version of the ME6000 Biomonitor System (Mega Electronics Ltd, Finland) to record the electrical signal from the skin of the research subjects. The signals were then bandpass filtered at 500-1000 Hz to display SKNA and at 1-150 Hz to display ECG data. The neuECG was recorded during baseline, stress 23, and recovery (5 minutes for each phase). The stress consisted of mental arithmetic stress induced by arithmetic involving the repeated serial subtraction of the number 13 from a 4-digit number for 5 minutes. The data were analyzed using customized software to determine the average SKNA (aSKNA [µV]) per digitized sample over the monitoring period 7. The research subjects were asked to lie down and rest for 10 minutes prior to SKNA recording to reduce bias due to motion artifacts and SNS interference.

Short-term HRV analysis was conducted for 5-minute intervals during each phase of the SKNA recording to facilitate comparison. The R peak of the QRS complex in each 5-minute window of the neuECG signal was automatically detected by the modified Pan Tompkins algorithm 24, and the RR interval was obtained beat by beat. We analyzed the time and frequency domains of the HRV using MATLAB (MathWorks, Inc., USA) 24,25.

### 2.3. Fecal Sample Collection

The fecal samples were frozen immediately after collection and delivered to the laboratory in a cooler bag within 24 hours. The samples were subsequently stored at -80 °C for up to 3 days before processing.

### 2.4. Fecal DNA Extraction and 16S rRNA Sequencing

Fecal DNA was extracted using a stool DNA extraction kit (Topgen Biotechnology Co., Ltd., Kaohsiung, Taiwan) as previously described 26. The concentration and quality of the DNA were assessed using a Colibri Microvolume spectrophotometer (Titertek Berthold, Pforzheim, Germany) before it was frozen at -20 °C to preserve its integrity.

The V3-V4 regions of the 16S rRNA gene in each sample were amplified using the primer pairs 341F (5'-TCGTCGGCAGCGTCAGATGTGTATAAGAGACAGCCTACGGGNGGCWGCAG-3') and 805R (5'-GTCTCGTGGGCTCGGAGATGTGTATAAGAGACAGGACTACHVGGGTATCTAATCC -3'). A library was prepared using the Illumina MiSeq platform, generating 2 × 300 bp reads. Raw sequence data were imported into QIIME2 27, where paired-end reads were merged and denoised into amplicon sequence variants (ASVs) using the DADA2 plugin 28, as previously described 26.

### 2.5. Statistical Analysis

Disease status (ACS vs. healthy control) was our primary comparison variable, with the baseline SKNA value as a continuous outcome. A priori sample size calculation, assuming a two-sided type I error of 5%, a standard deviation of 0.25 for SKNA, and a 1:1 case/control ratio, estimated a total of 28 participants (14 ACS and 14 controls) to achieve 80% statistical power for detecting an effect size of 0.28.

The significance of differences in the anthropometric data, clinical factors, laboratory results, SKNA parameters, and HRV parameters between the ACS and control groups was assessed using SPSS version 18.0 for Windows (SPSS Inc., Chicago, Illinois). The *P* values were calculated using the Mann‒Whitney *U* test for continuous data and the chi‒square test for categorical data. A *P* value of < 0.05 was considered to indicate statistical significance.

### 2.6. Bioinformatics

We employed the Chao 1 index and Faith's phylogenetic diversity to assess alpha diversity and compared differences between the ACS and control groups using Kruskal‒Wallis tests. For beta diversity, we performed an analysis of similarity (ANOSIM) and a permutational multivariate analysis of variance (PERMANOVA) with 999 permutations. These analyses were assessed using principal coordinate analyses (PCoAs) based on unweighted UniFrac measurements 29. Additionally, redundancy analysis was conducted to examine the relationships between microbial beta diversity and clinically relevant factors.

ASV taxonomy was assigned using the SciKit Learn-based approach 30, querying the SILVA reference database (release v138, trimmed to the V3-V4 region, L7 taxonomy) 31. Significantly different taxa between the ACS and control groups were identified using linear discriminant analysis effect size (LEfSe) with *P* < 0.05 (factorial Kruskal‒Wallis test) 32. To increase the stringency, the logarithmic linear discriminant analysis (LDA) score was set to 3. As extensively benchmarked in recent literature, traditional methods can inflate false positive rates in small cohorts. Therefore, ALDEx2 and ANCOM-BC, which are highly conservative and effectively control the False Discovery Rate (FDR) 33, were further employed to identify the genera that were discriminant between the two groups. Generalized linear models were used to explore the relationships between the targeted genera and autonomic parameters, with age and sex included as covariates.

Phylogenetic investigation of communities by reconstruction of observed states 2 (PICRUSt2) was utilized to predict functional differences between the microbial communities of the ACS and control groups based on the MetaCyc database 34.

## 3. Results

### 3.1. Clinical Characteristics

A total of 15 patients with ACS were included in this study, along with matched control subjects. The clinical characteristics are shown in Table 1. None of the anthropometric data, health habits, or clinical factors significantly differed between these groups. In terms of laboratory data, the ACS group had significantly elevated fasting glucose and HbA1c levels and markedly reduced HDL levels compared with those in the control group. The SKNA parameters during the baseline and recovery phases (SKNAb and SKNAr) were significantly greater in the ACS group. Additionally, the ACS group had significantly diminished SDNN values during the stress phase (SDNNs) for the HRV parameters.

Medication use differed substantially between the two groups, with ACS patients receiving multiple cardiovascular medications, whereas control participants had minimal medication exposure (Table S1).

### 3.2. Gut Microbiota Composition Differs between ACS Patients and Healthy Controls

The composition of gut microbiota in the ACS and control groups was determined using 16S rRNA gene sequencing. As depicted in Figure 1, a notable reduction in alpha diversity was observed in the ACS group, as evidenced by the Chao 1 index (*P* value < 0.001) and Faith's phylogenetic diversity (*P* value < 0.001). Distinct beta diversity clustering of the gut microbiota between the ACS and control groups was observed using unweighted UniFrac (ANOSIM: R = 0.702, *P* value = 0.001; PERMANOVA: pseudo-F = 9.573, *P* value = 0.001) or weighted unnormalized UniFrac measurements (ANOSIM: R = 0.123, *P* value = 0.008; PERMANOVA: pseudo-F = 2.860, *P* value = 0.011), as illustrated in Figure 2.

### 3.3. Associations between Clinically Relevant Variables and Microbial Beta Diversity

The association of clinically relevant variables with microbial beta diversity was further investigated by RDA. Fasting glucose was excluded from further analysis due to stress hyperglycemia in the ACS subjects despite significant differences between the ACS and control groups. To mitigate the potential accumulation of signals, the SKNA data were segregated into three distinct phases: baseline, stress, and recovery (Figures 3A to 3C). SKNAb, SKNAr, SDNNs, and HDL had the most potent influences (the longest arrow) on microbial beta diversity. The SKNAb and SKNAr were positively correlated with the ACS group during the baseline and recovery phases, respectively. Conversely, the SDNNs was negatively correlated with the ACS group during the stress phase. HDL levels were positively correlated with the control group in all three phases.

The results of the Mantel test further confirmed that SKNAb (*r* = 0.151, *P* value = 0.023), SDNNs (*r* = 0.249, *P* value = 0.002), and SKNAr (*r* = 0.209, *P* value = 0.007) were significantly related to the gut microbiota profiles during the baseline, stress, and recovery phases, respectively (Table 2). HDL levels were significantly associated with the dissimilarity of the gut microbiota in all three phases. No other clinical parameters were significantly related to microbial beta diversity.

### 3.4. Relative Abundances of Fecal Microbiota between the ACS and Control Groups

Relative differences in the abundances of fecal microbiota between the ACS and control groups were estimated by LEfSe, employing a logarithmic LDA score threshold of > 3. The phylum Desulfobacterota and the class Desulfovibrionia, as well as 3 orders, 6 families, 15 genera, and 19 species were enriched within the ACS group (Figure S1). Within the control group, enrichment was observed in the phylum Bacteroidota and the classes Actinobacteria and Bacteroidia, as well as in 3 orders, 7 families, 29 genera, and 45 species. Notably, the abundance of *Bifidobacterium bifidum*, which is classified as a lactic acid bacterium, increased throughout its taxonomic hierarchy to the class level in the control group.

To increase the stringency of discriminant taxa identification and rigorously control for FDRs in our cohort, a consensus approach utilizing three differential abundance algorithms (LEfSe, ALDEx2, and ANCOM-BC) was employed. A total of 12 genera were robustly identified as discriminant across all three methods (Figure 4A). Among them, *Eisenbergiella*, *Hungatella*, and Ruminococcaceae UBA1819 were significantly enriched in the ACS group. Conversely, the remaining genera exhibited a decreased abundance in the ACS group. This depletion included key members of the Prevotellaceae family (*Paraprevotella*, *Prevotella*, and *Prevotella*_9), as well as butyrate-producing bacteria *Lachnospira* and *Oscillibacter*. Additionally, an unexpected decrease in the opportunistic microbe *Haemophilus* was observed in the ACS group.

### 3.5. Links between the Gut Microbiome and the Clinical Characteristics of ACS

Because HDL-C, SKNAb, SDNNs, and SKNAr were significantly associated with the dissimilarity of the gut microbiome between the ACS and control groups, we proceeded to explore the correlations among the microbial relative abundances of the 12 genera that were identified as robust discriminant taxa by the consensus of the LEfSe, ALDEx2, and ANCOM-BC analyses. As shown in Figure 4B, *Hungatella* was negatively correlated with the SDNNs (*r* = -0.392, *P* value = 0.032) and positively correlated with the SKNAr (*r* = 0.441, *P* value = 0.015); notably, it also showed a marginally significant correlation with SKNAb (*r* = 0.358, *P* value = 0.052). Additionally, *Lachnospira* was positively correlated with SDNNs (*r* = 0.393, *P* value = 0.032). None of the other robust discriminant taxa were significantly correlated with the four clinical parameters. Based on the results of these highly stringent differential abundance analyses, we further analyzed the fixed effects to determine the correlation between SKNAb, SKNAr, and SDNNs and the abundance of the gut microbiota after controlling for age and sex (Figure 5A and B). The fixed effects of SKNAb and SKNAr on *Hungatella* were 0.66 (*P* < 0.001) and 5.757 (*P* < 0.001), respectively. The fixed effect of SDNNs on *Lachnospira* was 0.057 (*P* < 0.001).

### 3.6. Prediction of Microbial Functions

PICRUSt2 analysis revealed that 49 and 41 MetaCyc pathways were enriched in the ACS and control groups, respectively (Figure 6). In the ACS group, 12 and 10 pathways were significantly enriched for amino acid biosynthesis and nucleoside and nucleotide biosynthesis, respectively. Among them, 7 pathways related to the biosynthesis of branched-chain amino acids (BCAAs) were noted. With respect to the control group, 21 pathways were enriched under cofactor, prosthetic group, electron carrier, and vitamin biosynthesis, and 4 pathways were enriched under aromatic compound degradation.

## 4. Discussion

Although Meng et al. highlighted that the gut microbe-derived metabolite TMAO plays an important role in the development of VA by activating the cardiac SNS 16, our study is the first to demonstrate the interplay between the skin SNS and gut dysbiosis in ACS patients. Previously, we showed that SKNA was associated with VA in ACS patients, establishing it as a novel biomarker for ACS risk stratification 12. The current study further suggests a potential association between SKNA and gut microbiota profiles in ACS patients.

The value of SKNA was positively correlated with the relative abundance of *Hungatella*, a TMA producer (Figure 4 and Figure 5A). *Hungatella*, combined with *Eggerthella lenta*, has been reported to be involved in the conversion of L-carnitine to trimethylamine (TMA) 35. TMA is the diet-derived precursor of TMAO, the major metabolite of the microbiome correlated with the outcomes of ACS 14. Altered *Hungatella* abundance was linked to an increased incidence of unruptured intracranial aneurysms, an atherosclerotic cardiovascular disease 36. In two independent cross-sectional cohorts of patients with systolic heart failure (HF), increased *Hungatella* abundance was associated with HF 37. *Hungatella* was also negatively correlated with HDL-C levels in stroke patients 38 and positively correlated with IL-10 levels 39. Therefore, *Hungatella* is associated with cardiovascular disease and ACS. In addition, SGLT2i can reduce *Hungatella* abundance in individuals with type 2 diabetes 40. Furthermore, polyphenol-rich extracts can lower plasma TMAO levels in overweight and obese adults in association with *Hungatella* modulation 41, suggesting that modulation of *Hungatella* may be associated with cardiovascular risk. In addition to cardiovascular disease, *Hungatella* was also found to be more significantly enriched in patients with generalized anxiety disorder 42. Given that anxiety is thought to be correlated with high SNS status, the association between *Hungatella* and SNS is reasonable.

Type 2 diabetes, which is often linked with gut dysbiosis, is prevalent among patients with ACS and is recognized as a risk factor for the development of ACS. However, Emoto et al. compared the composition of the gut microbiome among coronary artery disease (CAD) patients, non-CAD controls who have coronary risk factors such as type 2 diabetes, and healthy volunteers and demonstrated that a change in the gut microbiota in the CAD group was not associated with type 2 diabetes 43. A key aspect of our study was the exclusion of patients with type 2 diabetes to clearly define the changes in the gut microbiota profiles specific to ACS. Compared with the matched controls, ACS patients exhibited a decrease in the abundance of butyrate-producing bacteria and an increase in the abundance of TMA-producing bacteria. These findings align with those of previous studies that included patients with type 2 diabetes 13,15, further supporting their potential involvement in ACS.

Predicted elevated production of BCAAs, including leucine, isoleucine, and valine, was observed in the microbial functional analysis using PICRUSt2 (Figure 6). Among the 12 pathways related to amino acid biosynthesis that were enriched in ACS subjects, five were associated with isoleucine biosynthesis, one with valine biosynthesis, and one with overall BCAA biosynthesis. Previous studies have identified increased plasma BCAA concentrations in patients with heart failure or coronary artery disease that can predict adverse outcomes 17-19. In addition to impaired cardiac BCAA catabolism, abnormal BCAA accumulation may originate from the gut microbiota 20. Additionally, BCAA accumulation has been shown to exacerbate microglia-induced neuroinflammation in a rat model of ischemia/reperfusion 44. These findings may reflect a potential link between the microbiota and SKNA in ACS, as addressed in this study.

This study has several limitations. First, the sample size was relatively small due to the rigorous selection process, including strict exclusion criteria to minimize confounding factors affecting gut microbiota. Although this approach improves internal validity, it may limit generalizability and increase the risk of statistical instability in high-dimensional microbiome analyses. Therefore, the findings should be interpreted as exploratory and require validation in larger independent cohorts. Second, the use of the V3-V4 region for 16S rRNA gene sequencing limited taxonomic resolution to the genus level in some cases. Third, we did not measure TMAO or BCAA levels in the subjects, preventing direct evaluation of their relationships with the gut microbiota and SKNA. Thus, functional interpretations based on PICRUSt2 remain speculative and require validation using targeted metabolomics, metagenomics, or experimental studies (e.g., in vitro or animal models). Fourth, this study was not designed to evaluate the incremental diagnostic or prognostic value of SKNA beyond established HRV parameters; therefore, its clinical implications remain preliminary. Finally, residual confounding cannot be excluded. Differences in metabolic parameters (fasting glucose, HbA1c, and HDL) and other unmeasured factors, such as lifestyle and host metabolic status, may have influenced microbial composition. In addition, the relatively low correlation coefficients suggest that the observed associations are likely multifactorial and should be interpreted with caution. Due to the limited sample size and the relatively large number of potential metabolic and medication-related covariates, multivariable adjustment was not undertaken, as it would likely result in model overfitting and unstable estimates.

In conclusion, this study is the first to demonstrate an association between SKNA and gut microbial profiles in ACS patients. By applying stringent selection criteria to exclude patients with type 2 diabetes, we identified specific patterns in patients with ACS, including alterations in butyrate-producing bacteria, TMA-producing bacteria, and predicted microbial BCAA biosynthesis pathways. These findings provide preliminary evidence of a link between SKNA and gut microbiota in ACS and may contribute to a better understanding of the neurocardiac-microbiota axis. Further studies with larger cohorts and mechanistic validation are required to clarify the clinical relevance of SKNA in ACS.

## Supplementary Material

Supplementary figures and tables.

## Figures and Tables

**Figure 1:**
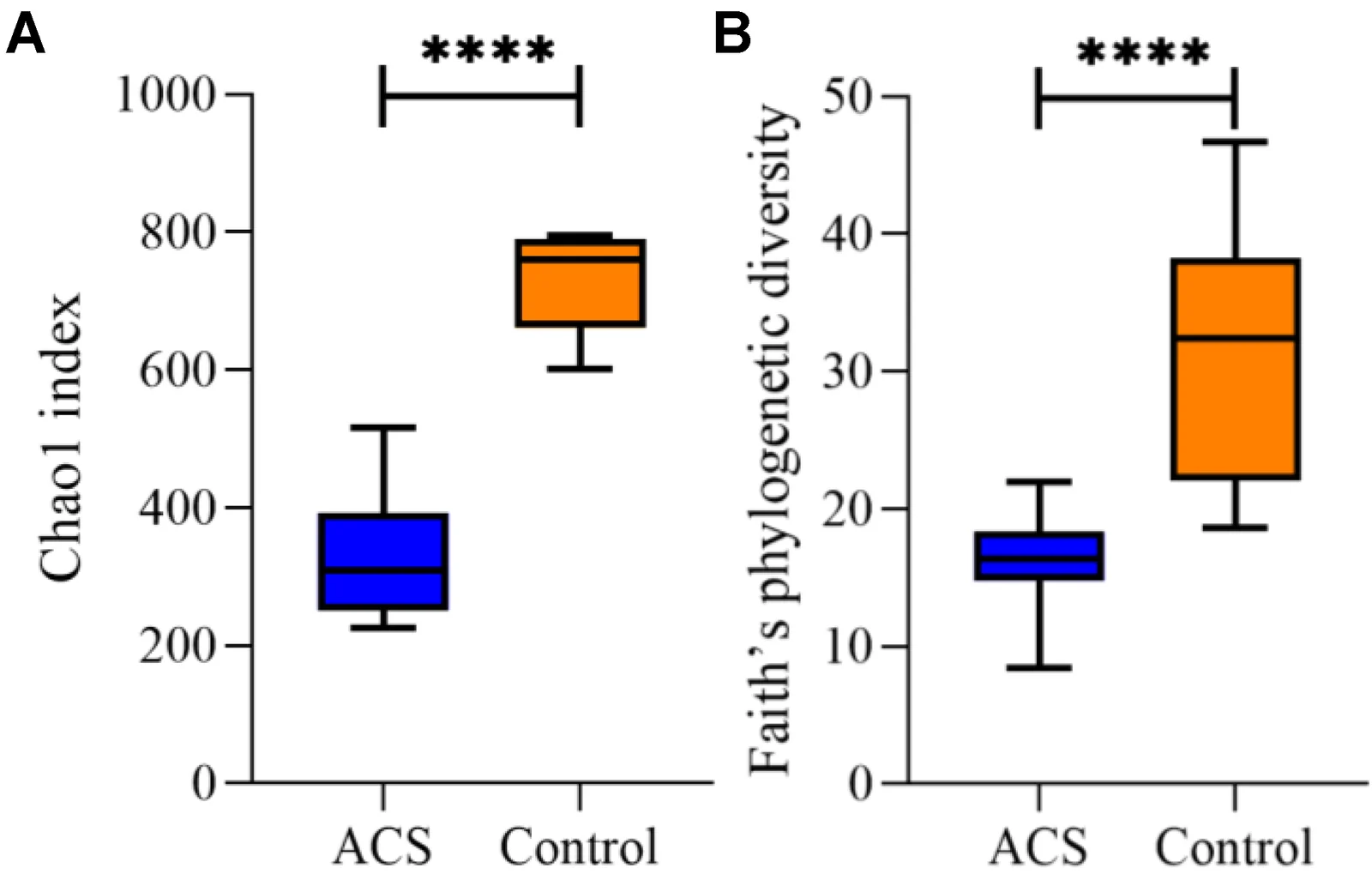
Comparison of the alpha diversity between the ACS and control groups. (A) The median Chao1 index was 309.00 (25th-75th percentile: 257.15-383.27) in the ACS group and 760.38 (671.15-783.42) in the control group. (B) The median Faith's phylogenetic diversity was 16.39 (25th-75th percentile: 14.81-18.20) in the ACS group and 32.46 (23.73-37.83) in the control group.

**Figure 2:**
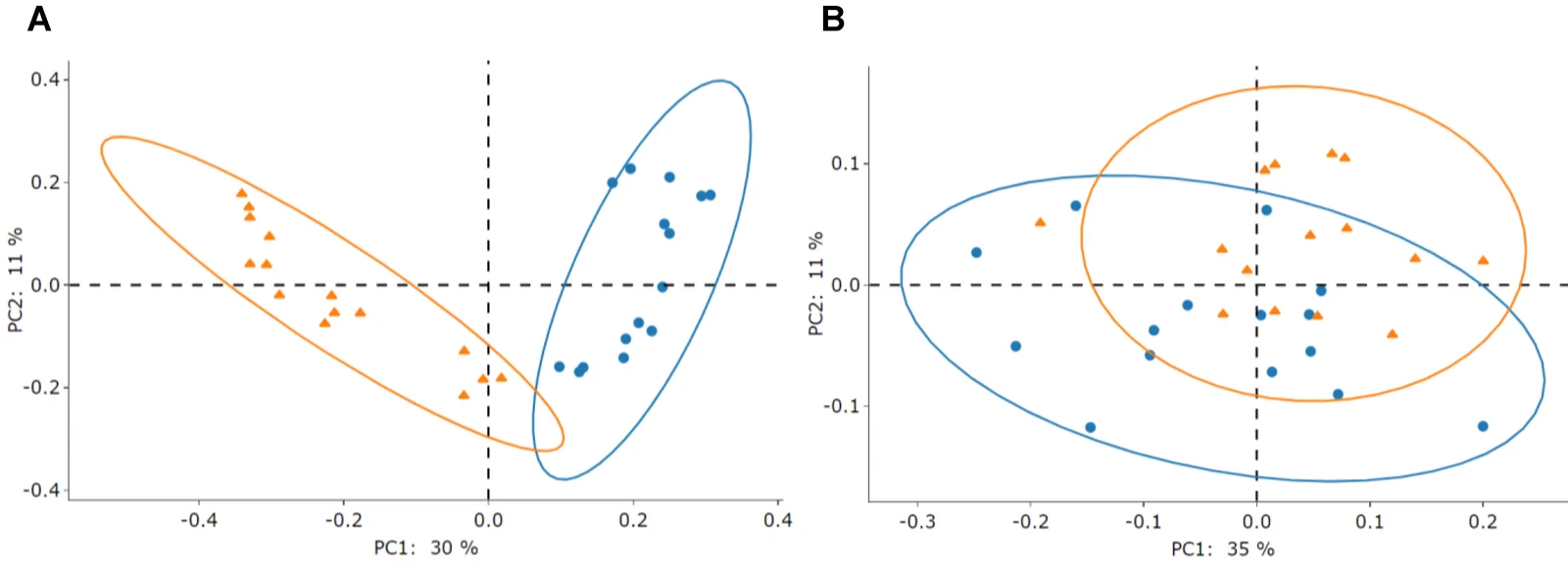
Beta diversity clustering of the gut microbiota between the ACS and control groups. (A) Unweighted UniFrac measurements. (B) Weighted unnormalized UniFrac measurements. The blue circle indicates the ACS group, and the orange triangle indicates the control group.

**Figure 3:**
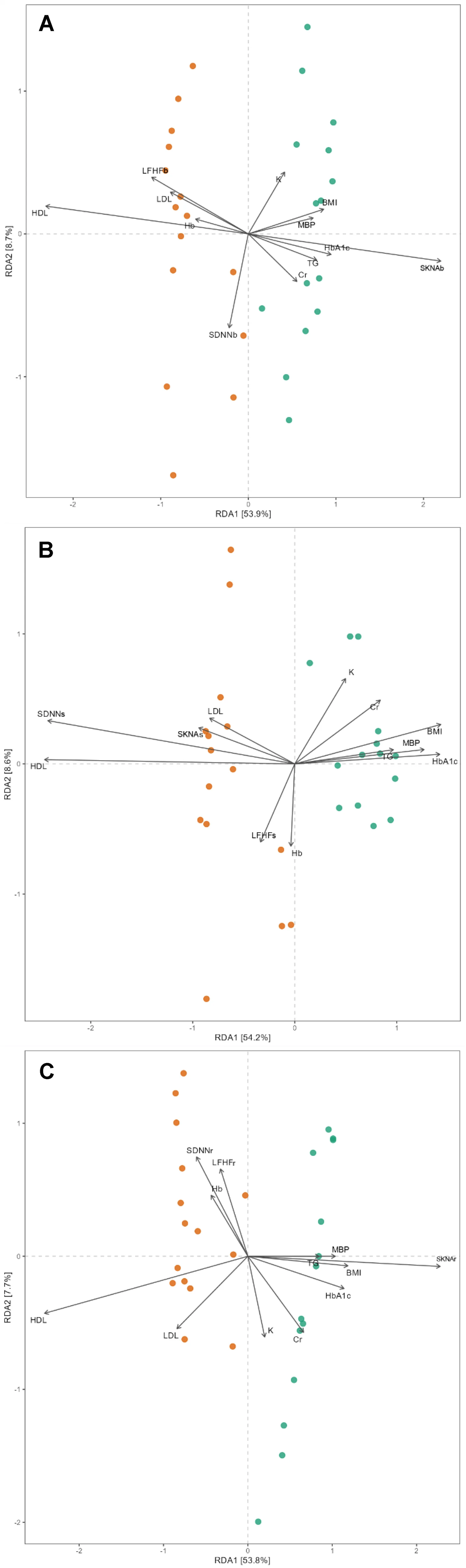
Comparison of clinically relevant factors influencing microbial beta diversity between the ACS and control groups. The clinical confounding factors of BMI (body mass index), Cr (creatinine), Hb (hemoglobin), HbA1c, HDL (high-density lipoprotein), K (potassium), LDL (low-density lipoprotein), LF/HF (low frequency power/high frequency power), MBP (mean blood pressure), SDNN (standard deviation of normal to normal R wave) and SKNA (skin sympathetic nerve activity) were examined by redundancy analysis (RDA) based on unweighted UniFrac measurements. (A) Baseline phase. (B) Stress phase. (C) Recovery phase. The green spot indicates the subjects belonging to the ACS group, and the orange spot indicates the subjects belonging to the control group.

**Figure 4:**
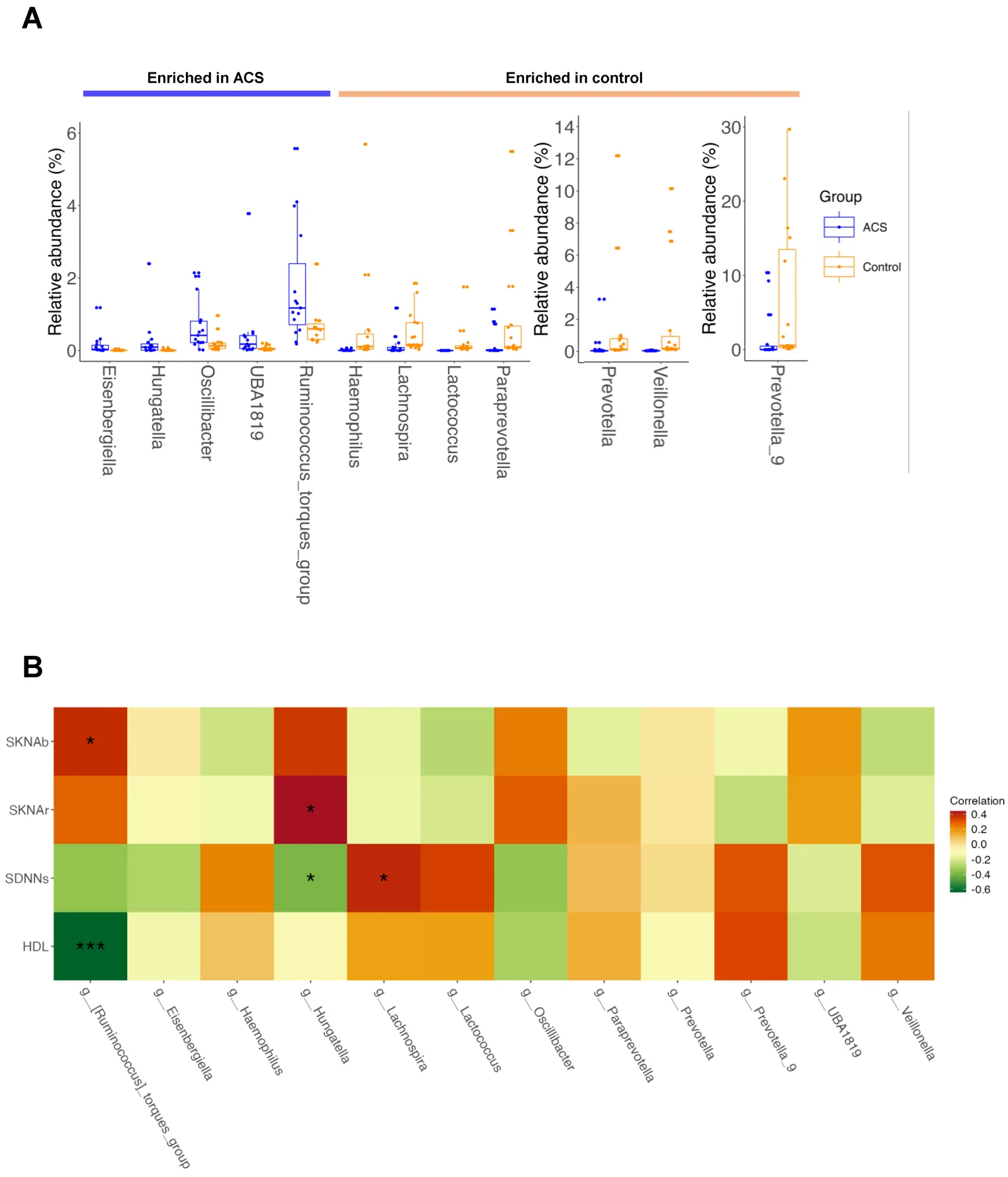
The 12 genera identified across LEfSe, ALDEx2, and ANCOM-BC. (A) The relative abundances. (B) Correlations with SKNAb, SKNAr, SDNNs and HDL.

**Figure 5:**
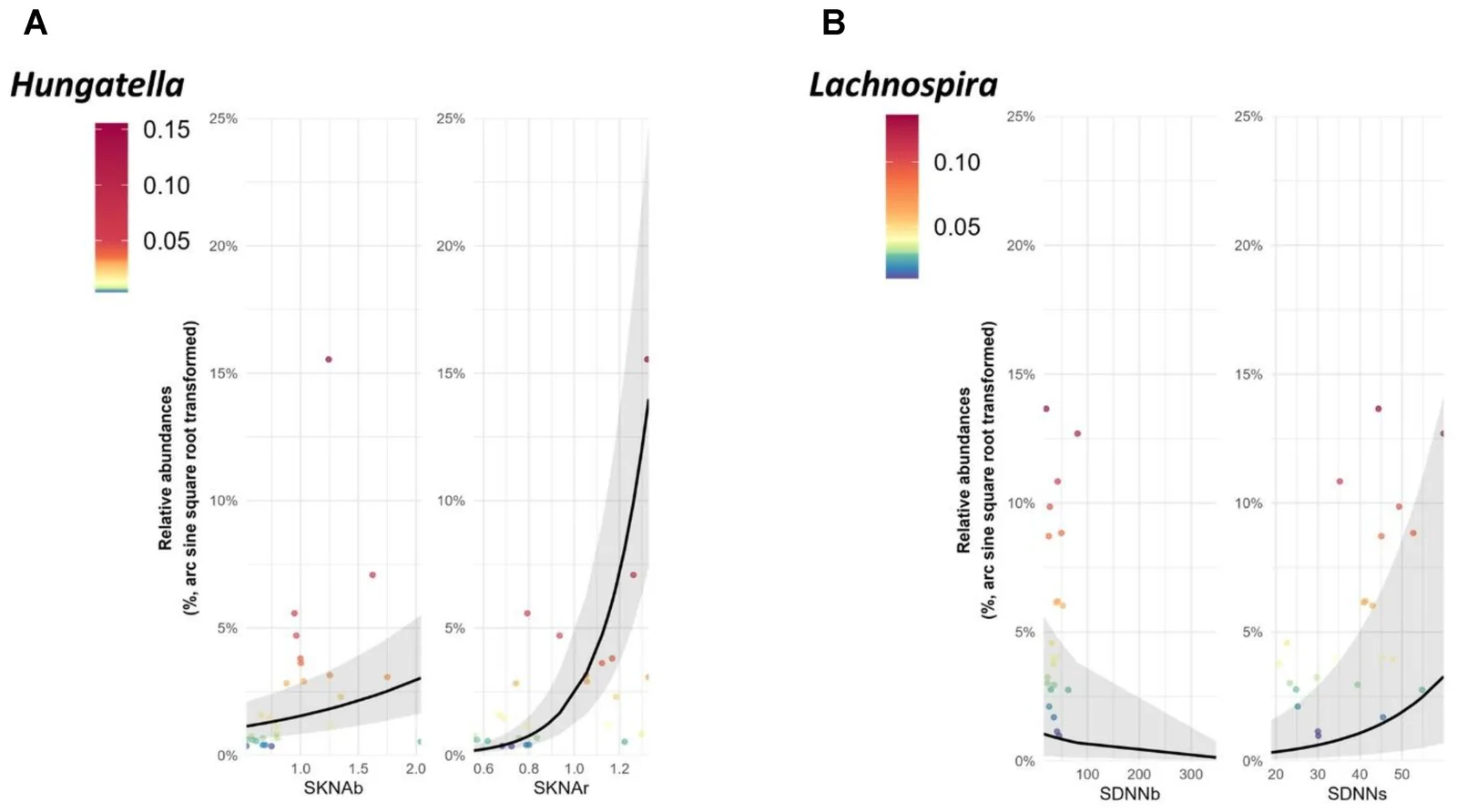
Fixed effects after controlling for age and sex to determine the correlation between the microbiota and the results of the autonomic survey. (A) The correlation between *Hungatella* and SKNA. (B) The correlation between *Lachnospira* and the SDNN.

**Figure 6:**
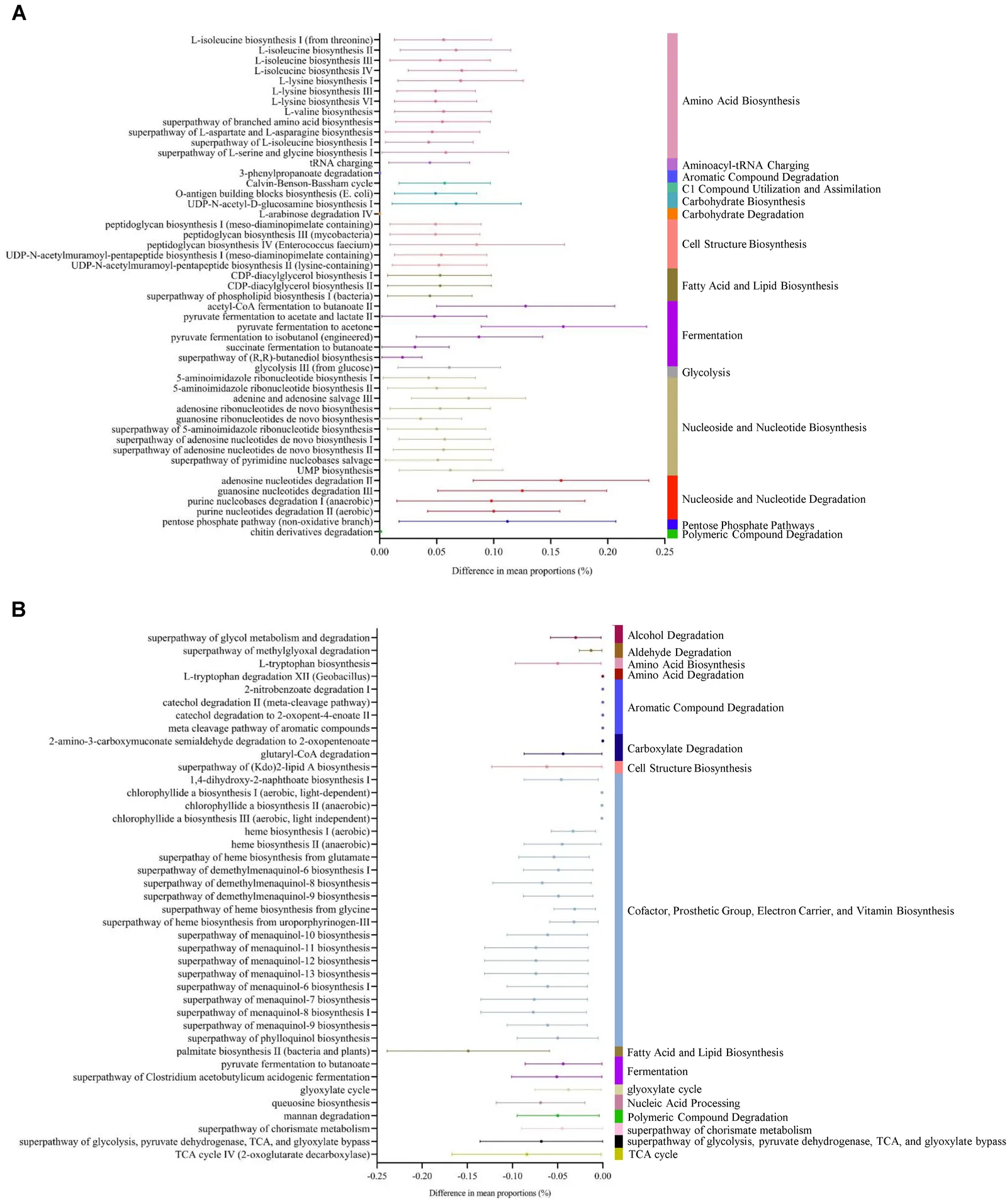
Comparison of PICRUSt2-predicted MetaCyc pathways between the ACS and control groups. (A) Pathways enriched in the ACS group. (B) Pathways enriched in the control group.

**Table 1:** Characteristics of the ACS and control groups.

	ACS group (n = 15)	Control group (n = 15)	*P* value
Anthropometric data			
Sex, male, n (%)	7 (46.67)	8 (53.33)	0.715
Age, y	64.00 (60.00-68.00)	63.00 (60.50-68.50)	0.868
Body mass index, kg/m^2^	24.70 (21.75-27.20)	22.40 (21.50-23.80)	0.206
Clinical factors			
Dyslipidemia, n (%)	7 (46.67)	7 (46.67)	1.000
Hypertension, n (%)	9 (60.00)	8 (53.33)	0.713
Smoke, n (%)	0 (0.0)	4 (26.7)	0.043
Alcohol, n (%)	2 (12.5)	1 (6.7)	1
Betel Nut, n (%)	0(0.0)	0(0.0)	1
Systolic BP, mmHg	139.00 (127.00-157.50)	137.00 (120.00-141.00)	0.407
Diastolic BP, mmHg	86.00 (71.00-91.00)	79.00 (71.00-87.00)	0.561
Mean BP, mmHg	107.00 (91.84-111.00)	95.67 (89.00-104.50)	0.330
Laboratory data			
Fasting glucose, mg/dL	109.00 (101.50-131.00)	83.00 (78.50-94.00)	**< 0.001**
HbA1c, %	5.60 (5.5-5.85)	5.30 (5.10-5.60)	**0.029**
Total cholesterol, mg/dL	181.00 (151.50-198.50)	215.00 (179.50-228.00)	0.059
Triglycerides, mg/dL	107.00 (67.50-162.50)	83.00 (75.00-121.50)	0.520
HDL, mg/dL	45.20 (30.75-50.90)	61.20 (53.40-66.15)	**< 0.001**
LDL, mg/dL	120.50 (72.25-130.75)	124.90 (101.35-139.50)	0.325
Hemoglobin level, g/dL	13.30 (12.25-14.40)	14.10 (13.25-14.65)	0.280
Hematocrit, %	40.20 (35.15-44.15)	43.30 (40.85-44.20)	0.105
Creatinine, mg/dL	0.81 (0.66-0.98)	0.72 (0.64-0.98)	0.756
eGFR, mL/min/1.73m^2^	80.96 (72.90-96.31)	93.00 (81.00-106.50)	0.267
K, mmol/L	3.60 (3.50-3.80)	3.60 (3.45-3.70)	0.391
SKNA parameters			
SKNAb, μV	1.02 (0.96-1.31)	0.69 (0.60-0.76)	**< 0.001**
SKNAs, μV	1.39 (1.27-1.58)	1.37 (1.20-1.41)	0.407
SKNAr, μV	1.12 (0.91-1.20)	0.72 (0.64-0.80)	**< 0.001**
HRV parameters			
SDNNb, ms	29.20 (25.00-41.90)	35.20 (30.95-4 5.00)	0.351
RMSSDb, ms	18.50 (11.6-28.20)	26.30 (16.35-29.65)	0.507
LFb	0.46 (0.41-0.62)	0.61 (0.52-0.69)	0.101
HFb	0.54 (0.38-0.59)	0.39 (0.31-0.48)	0.101
LF/HFb	0.87 (0.70-1.78)	1.54 (1.1-2.24)	0.106
SDNNs, ms	27.30 (23.82-31.68)	45.10 (40.15-48.60)	**< 0.001**
RMSSDs, ms	16.70 (14.15-21.18)	23.50 (21.25-26.35)	0.102
LFs	0.69 (0.63-0.76)	0.71 (0.65-0.82)	0.631
HFs	0.31 (0.24-0.37)	0.29 (0.18-0.35)	0.631
LF/HFs	4.49 (1.71-3.19)	2.47 (1.85-4.49)	0.652
SDNNr, ms	33.65 (22.95-49.15)	37.50 (30.75-42.90)	0.395
RMSSDr, ms	17.85 (10.13-30.13)	22.60 (17.55-26.65)	0.451
LFr	0.59 (0.55-0.71)	0.61 (0.51-0.74)	0.880
HFr	0.41 (0.29-0.45)	0.39 (0.26-0.49)	0.880
LF/HFr	1.47 (1.21-2.49)	1.55 (1.05-2.96)	0.880

Note: Data are presented as medians (interquartile ranges, 25th-75th percentiles). The b, s, and r values noted after the SKNA and HPV parameters indicate the baseline, stress, and recovery phases, respectively. Abbreviations: BP, blood pressure; eGFR, estimated glomerular filtration rate; HbA1c, glycated hemoglobin; HDL, high-density lipoprotein; HF, high-frequency power; HPV, heart rate variability; LDL, low-density lipoprotein; LF, low-frequency power; RMSSD, root mean square of the successive differences; SDNN, standard deviation of normal to normal R wave interval; SKNA, skin sympathetic nerve activity.

**Table 2:** Evaluation of the relationships between clinically relevant factors and gut microbial profiles by the Mantel test.

	Phase					
	Baseline		Stress		Recovery	
	Correlation coefficient	*P* value	Correlation coefficient	*P* value	Correlation coefficient	*P* value
SKNA	0.151	**0.023**	-0.014	0.522	0.209	**0.007**
SDNN	-0.028	0.603	0.249	**0.002**	-0.090	0.942
HDL	0.273	**0.003**	0.235	**0.002**	0.262	**0.002**

Abbreviations: HDL, high-density lipoprotein; SDNN, standard deviation of normal to normal R wave interval; SKNA, skin sympathetic nerve activity.
