# Supplementary material for: Interplay between Skin Sympathetic Nerve Activity and Gut Microbiota, the Neurocardiac Axis in Acute Coronary Syndrome
Source: Int J Med Sci. 2026 Aug 13;23(10):3090–100. doi: 10.7150/ijms.126417 (PMC13617496; doi:10.7150/ijms.126417)
Supplement: Supplementary file 1 [file ijmsv23p3090s1.pdf]

TABLE S1 Key Medications: ACS vs. Control

Medication class	ACS group (n = 15)	Control group (n = 15)
Antiplatelet agents	15	0
Anticoagulants	15	0
ACEi/ARB	11	0
Beta-blockers	11	0
Statins	13	0
Diuretics	4	0
Vasopressors	3	0
Other non-cardiovascular medications	0	2

Antiplatelet agents include aspirin, clopidogrel, ticagrelor, and tirofiban. Anticoagulants include low-molecular-weight heparin, unfractionated heparin, factor Xa inhibitors, and non-vitamin K antagonist oral anticoagulants. Statins include atorvastatin and rosuvastatin.

Angiotensin-converting enzyme inhibitors, ACEi; angiotensin receptor blockers, ARB.

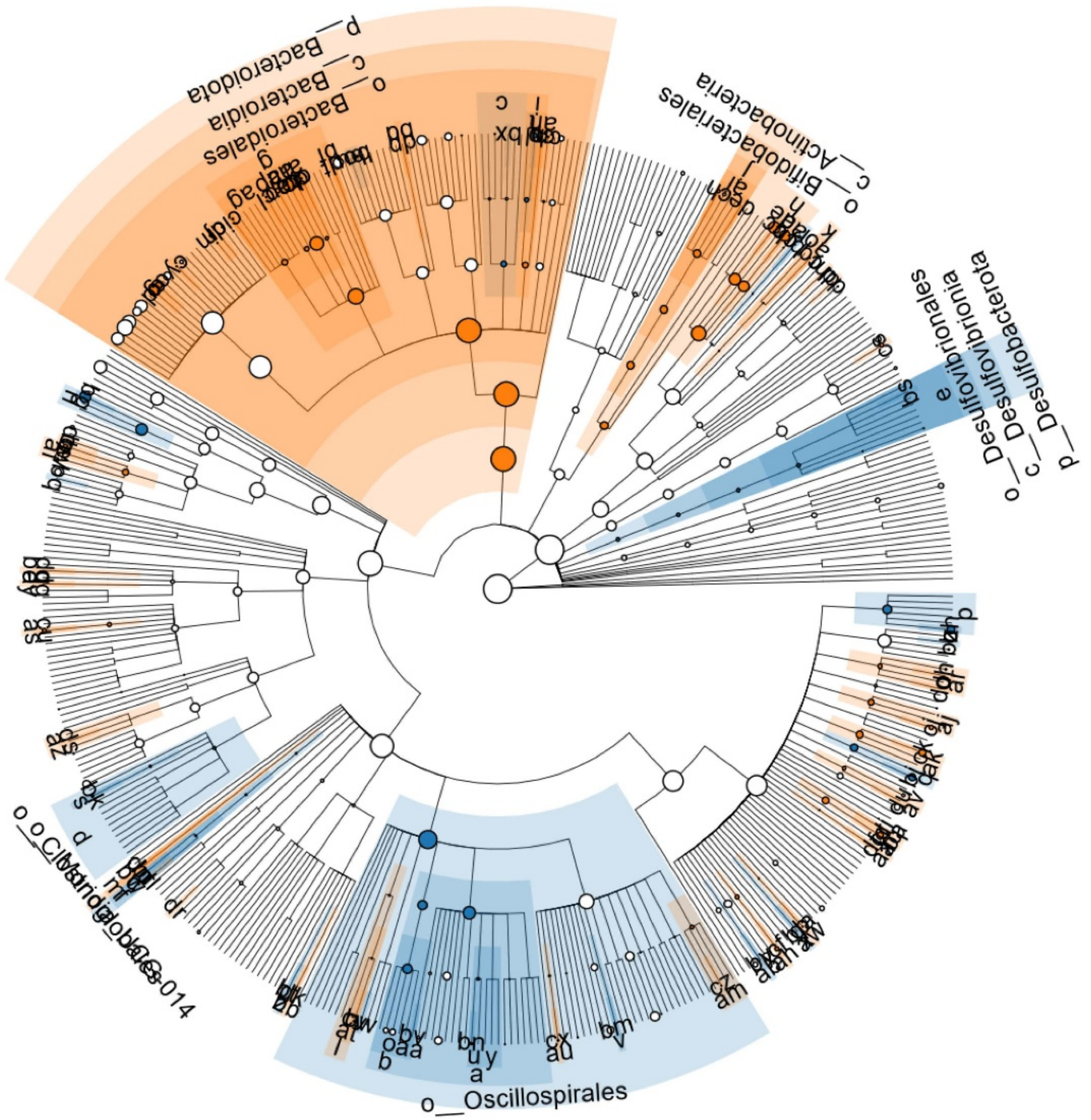

Figure S1. LEfSe analysis of the differences in the relative abundances of microbiota between the ACS and control groups.

Blue and orange indicate the significantly enriched microbiota in the ACS and control groups, respectively. The circles of cladogram from inside to outside represent the phylum, class, order, family, genus, and species.

AMI
Control

a: f__Oscillospiraceae
b: f__[Eubacterium]_coprostanoligenes_group
c: f__Marinifilaceae
d: f__Lactobacillaceae
e: f__Desulfovibrionaceae
f: f__Clostridia_UCG-014
g: f__Prevotellaceae
h: f__Enterobacteriaceae
i: f__Muribaculaceae
j: f__Bifidobacteriaceae
k: f__Pasteurellaceae
l: f__Butyricicoccaceae
m: f__Monoglobaceae
n: g__Megamonas
o: g__[Eubacterium]_coprostanoligenes_group
p: g__Lachnoclostridium
q: g__[Ruminococcus]_torques_group
r: g__Succinivibrio
s: g__Ligilactobacillus
t: g__Muribaculaceae
u: g__Oscillibacter
v: g__UBA1819
w: g__Clostridia_UCG-014
x: g__Hungatella
y: g__Colidextribacter
z: g__Peptoclostridium
aa: g__Family_Oscillospiraceae sp.
ab: g__Eisenbergiella
ac: g__Prevotella_9
ad: g__Escherichia-Shigella
ae: g__Klebsiella
af: g__Veillonella
ag: g__Prevotella
ah: g__Fusicatenibacter
ai: g__Bifidobacterium
aj: g__Roseburia
ak: g__Anaerostipes
al: g__Paraprevotella
am: g__Ruminococcus
an: g__CAG-873
ao: g__Haemophilus
ap: g__Prevotellaceae_NK3B31_group
aq: g__[Eubacterium]_hallii_group
ar: g__Coprococcus
as: g__Holdemanella
at: g__Butyricicoccus
au: g__CAG-352
av: g__Agathobacter
aw: g__Lachnospira
ax: g__[Eubacterium]_eligens_group
ay: g__Erysipelotrichaceae_UCG-003
az: g__Lactococcus
ba: g__[Eubacterium]_ruminantium_group
bb: g__Romboutsia
bc: g__Monoglobus
bd: g__Rikenellaceae_RC9_gut_group
be: g__Catenibacterium
bf: g__Family_Prevotellaceae sp.
bg: s__Genus_Megamonas sp.
bh: s__Genus_Lachnoclostridium sp.
bi: s__Genus_[Ruminococcus]_torques_group sp.
bj: s__Genus_Succinivibrio sp.
bk: s__Lactobacillus_salivarius
bl: s__Genus_Muribaculaceae sp.
bm: s__Genus_UBA1819 sp.
bn: s__Genus_Oscillibacter sp.
bo: s__Alistipes_shahii
bp: s__Genus_Hungatella sp.
bq: s__Megasphaera_hexanoica
br: s__Clostridia_UCG-014_gut_metagenome
bs: s__Desulfovibrio_piger
bt: s__Genus_Peptoclostridium sp.
bu: s__Alistipes_sp.
bv: s__Family_Oscillospiraceae sp.
bw: s__Genus_UCG-010 sp.
bx: s__Butyricimonas_virosa
by: s__Genus_Eisenbergiella sp.
bz: s__Blautia_sp.
ca: s__Genus_Prevotella_9 sp.
cb: s__Genus_Escherichia-Shigella sp.
cc: s__Genus_Klebsiella sp.
cd: s__Bacteroides_plebeius
ce: s__Genus_Veillonella sp.
cf: s__Genus_Fusicatenibacter sp.
cg: s__Bacteroides_ovatus
ch: s__Genus_Bifidobacterium sp.
ci: s__Prevotella_stercorea
cj: s__Genus_Roseburia sp.
ck: s__Genus_Anaerostipes sp.
cl: s__Genus_Prevotella sp.
cm: s__Bacteroides_coprocola
cn: s__Genus_Coprobacter sp.
co: s__Genus_CAG-873 sp.
cp: s__Genus_Prevotellaceae_NK3B31_group sp.
cq: s__Genus_Haemophilus sp.
cr: s__Genus_[Eubacterium]_hallii_group sp.
cs: s__Fusobacterium_varium
ct: s__Genus_Paraprevotella sp.
cu: s__Genus_Holdemanella sp.
cv: s__Genus_Butyricicoccus sp.
cw: s__Veillonella_atypica
cx: s__Genus_CAG-352 sp.
cy: s__Bacteroides_intestinalis
cz: s__Ruminococcus_bicirculans
da: s__Genus_Lachnospira sp.
db: s__Genus_Dialister sp.
dc: s__Paraprevotella_xylaniphila
dd: s__Sutterella_gut_metagenome
de: s__Bifidobacterium_bifidum
df: s__Genus_[Eubacterium]_eligens_group sp.
dg: s__Genus_Erysipelotrichaceae_UCG-003 sp.
dh: s__Coprococcus_unidentified
di: s__Genus_[Eubacterium]_ruminantium_group sp.
dj: s__Bacteroides_clarus
dk: s__Genus_Romboutsia sp.
dl: s__Genus_Clostridia_UCG-014 sp.
dm: s__Sutterella_wadsworthensis
dn: s__Genus_Monoglobus sp.
do: s__Coprococcus_eutactus
dp: s__Genus_Rikenellaceae_RC9_gut_group sp.
dq: s__Genus_Catenibacterium sp.
dr: s__Clostridium_perfringens
ds: s__Lactococcus_garvieae
dt: s__Family_Prevotellaceae sp.
du: s__Eubacterium_ramulus
